# Supplementary figures and images for: Gamma radiation-induced molecular toxicity and effects on pluripotent stem cells of the radiosensitive conifer Norway spruce (Picea abies)
Source: Planta. 2025 Sep 17;262(5):102. doi: 10.1007/s00425-025-04819-6 (PMC12443939; doi:10.1007/s00425-025-04819-6)

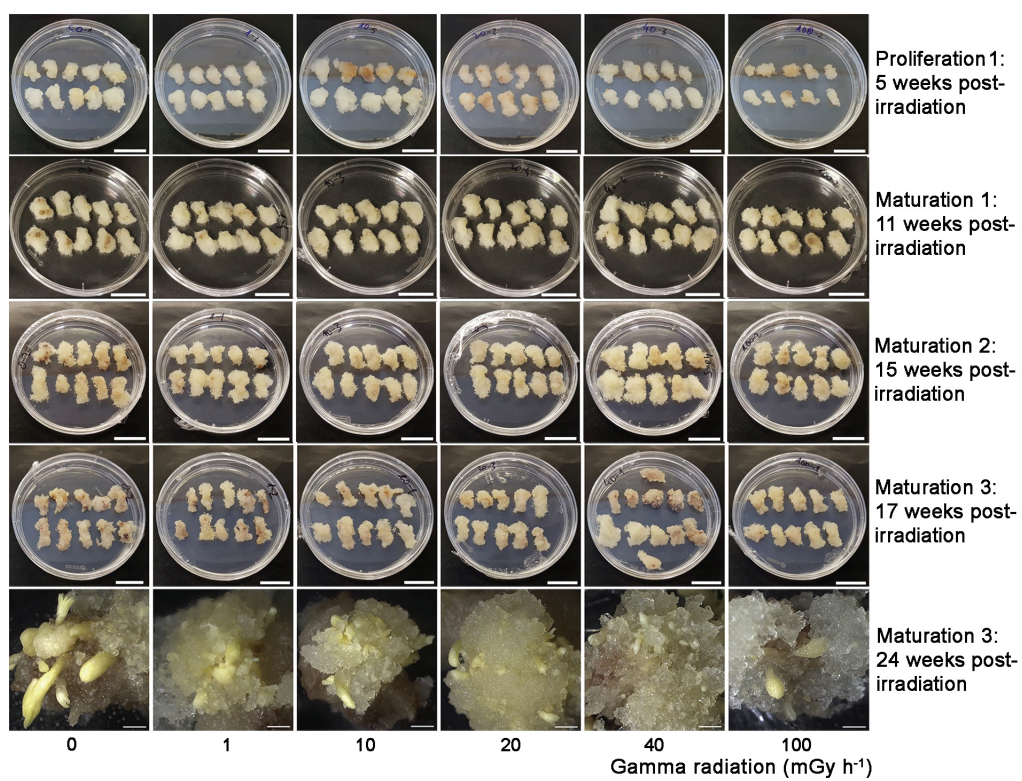

Figure S1. Bhattacharjee and Lee et al., 2025. □

Supplement: Supplementary file 1 — Supplementary file1 (PDF 11949 kb) [file 425_2025_4819_MOESM1_ESM.pdf]

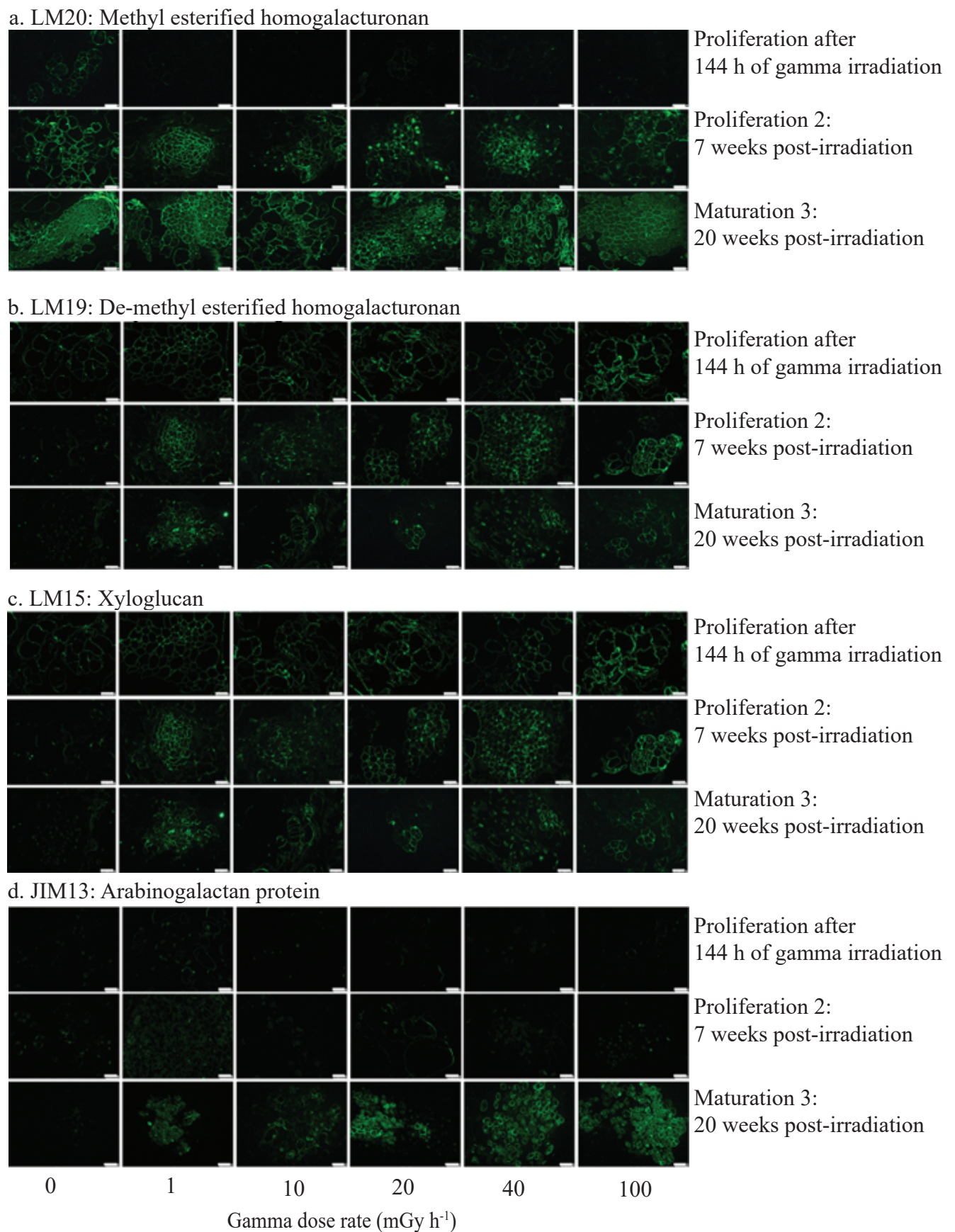

Figure S2. Bhattacharjee and Lee et al., 2025.

Supplement: Supplementary file 3 — Supplementary file3 (PDF 1907 kb) [file 425_2025_4819_MOESM3_ESM.pdf]

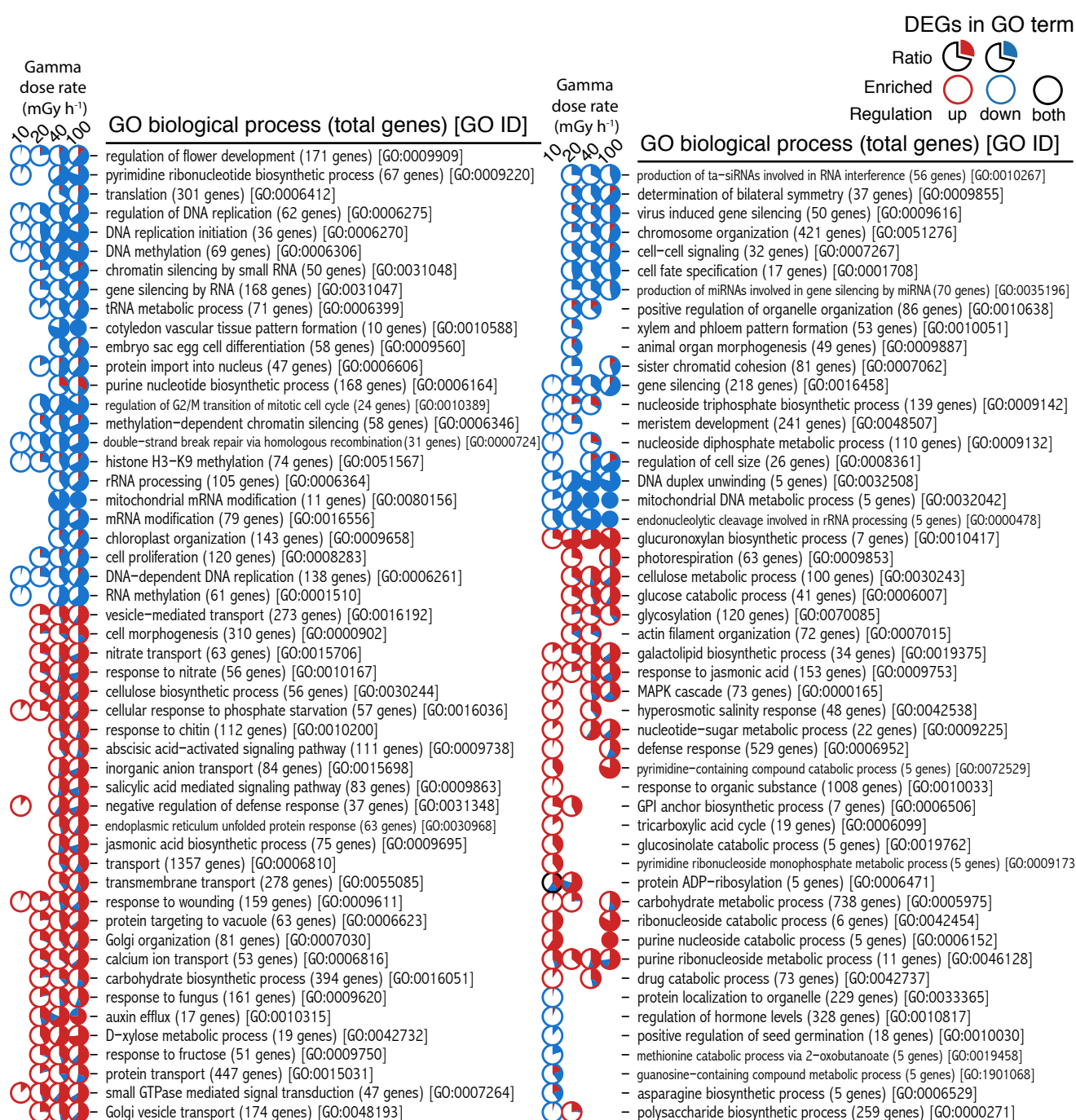

Figure S3. Bhattacharjee, Lee et al., 2025

Supplement: Supplementary file 5 — Supplementary file5 (PDF 1617 kb) [file 425_2025_4819_MOESM5_ESM.pdf]

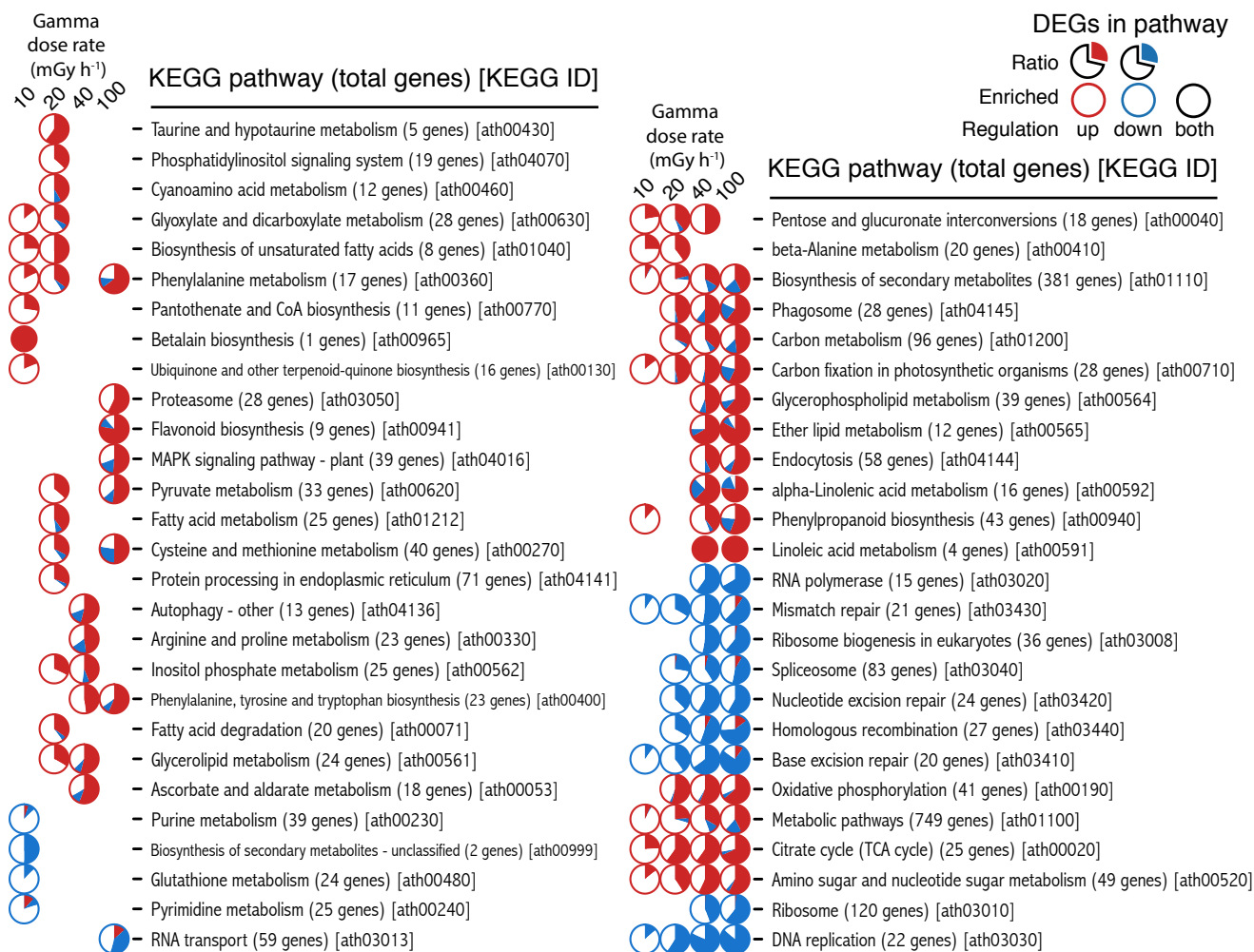

Figure S4. Bhattacharjee, Lee et al., 2025

Supplement: Supplementary file 7 — Supplementary file7 (PDF 932 kb) [file 425_2025_4819_MOESM7_ESM.pdf]
