# Supplementary material for: Gamma radiation-induced molecular toxicity and effects on pluripotent stem cells of the radiosensitive conifer Norway spruce (Picea abies)
Source: Planta. 2025 Sep 17;262(5):102. doi: 10.1007/s00425-025-04819-6 (PMC12443939; doi:10.1007/s00425-025-04819-6)
Supplement: Supplementary file 2 — Supplementary file2 (PDF 11976 kb) [file 425_2025_4819_MOESM2_ESM.pdf]

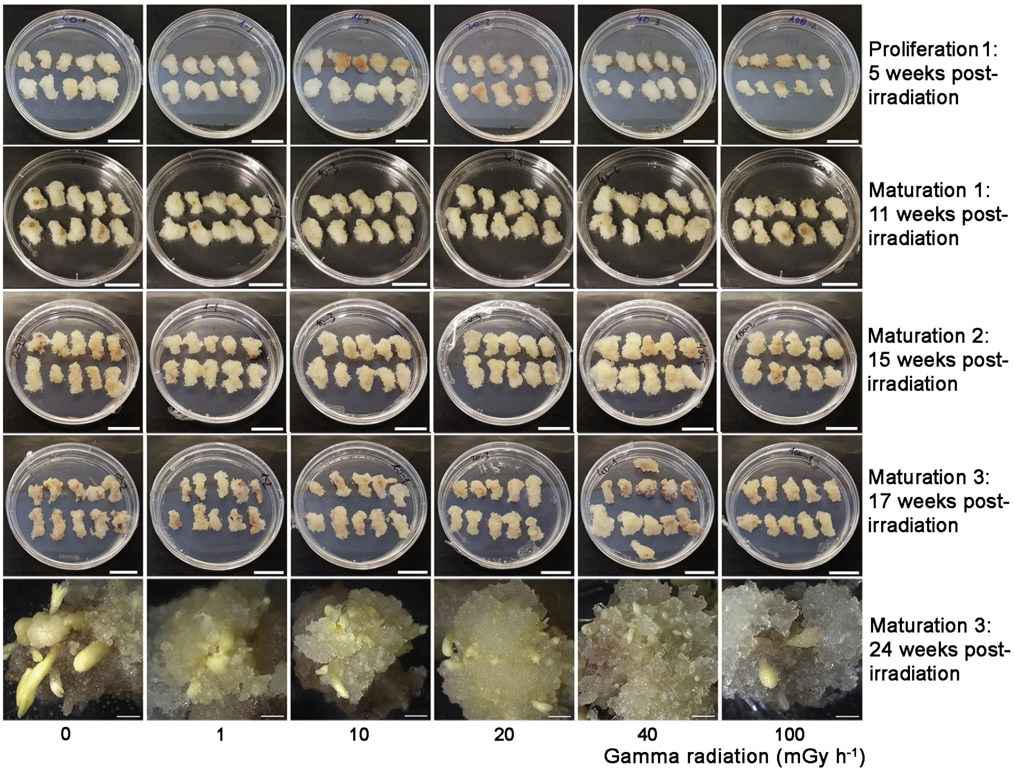

Fig. S1 Post-irradiation development of clonal stem cell aggregates and somatic embryos of Norway spruce following 144 h of gamma irradiation. Scale bars: 2:cm (4 upper panels) and 2 mm (lowest panel). Representative cell aggregates out of totally 40-60 irradiated ones per dose rates are shown.

Figure S1. Bhattacharjee and Lee et al.,2025.
