# Supplementary material for: Gamma radiation-induced molecular toxicity and effects on pluripotent stem cells of the radiosensitive conifer Norway spruce (Picea abies)
Source: Planta. 2025 Sep 17;262(5):102. doi: 10.1007/s00425-025-04819-6 (PMC12443939; doi:10.1007/s00425-025-04819-6)
Supplement: Supplementary file 4 — Supplementary file4 (PDF 126 kb) [file 425_2025_4819_MOESM4_ESM.pdf]

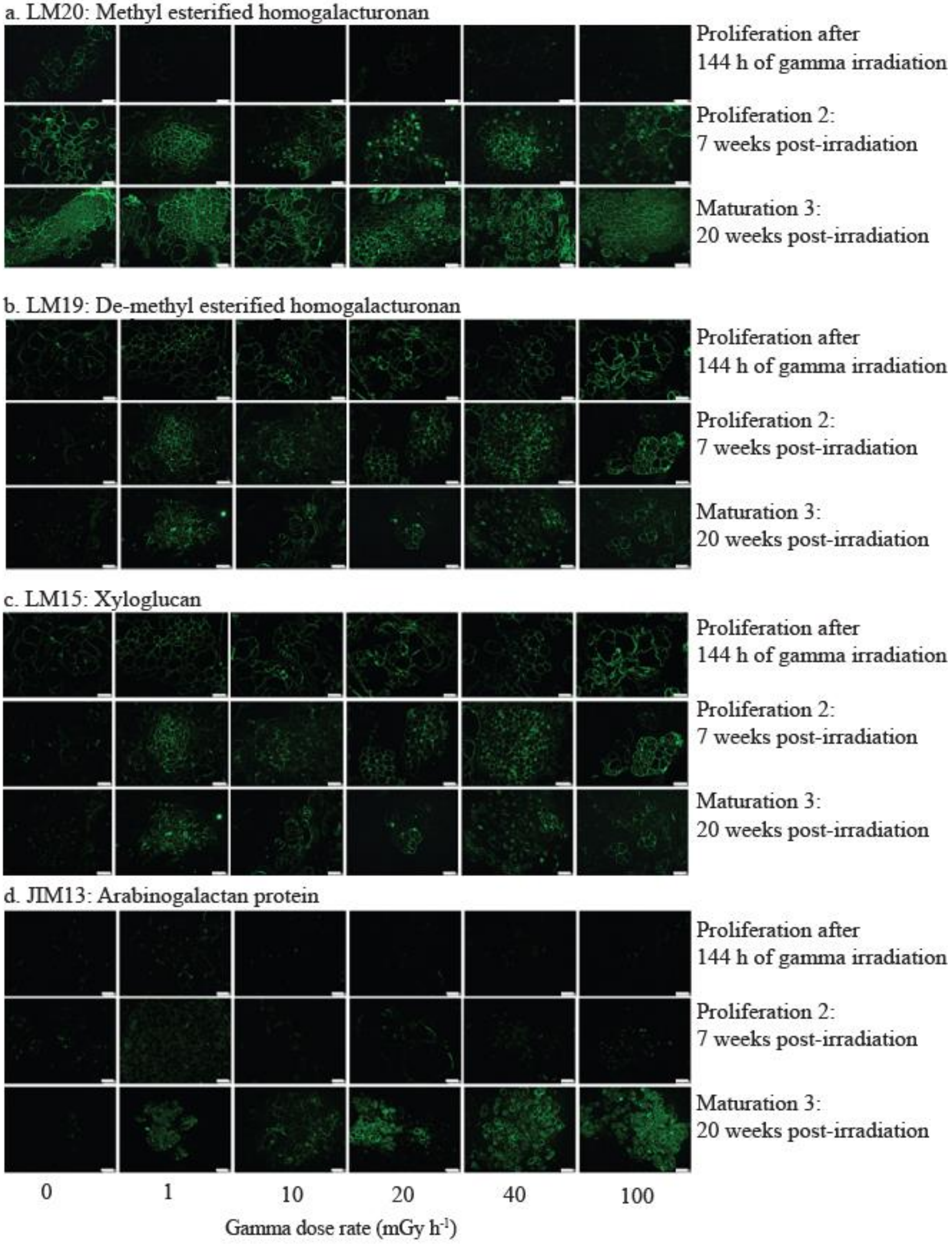

Figure S2. Bhattacharjee and Lee et al.,2025.

Fig. S2 Micrographs of indirect immunofluorescence detection of cell wall components in proliferating stem cell aggregates of Norway spruce at the end of 144 h of gamma irradiation and during post-irradiation stages. Longitudinal 1 μm thick sections were probed with the monoclonal antibodies. Sections were made from 3 plants per gamma radiation dose rate. Scale bars: 50 μm.
