# Supplementary material for: Gamma radiation-induced molecular toxicity and effects on pluripotent stem cells of the radiosensitive conifer Norway spruce (Picea abies)
Source: Planta. 2025 Sep 17;262(5):102. doi: 10.1007/s00425-025-04819-6 (PMC12443939; doi:10.1007/s00425-025-04819-6)
Supplement: Supplementary file 6 — Supplementary file6 (PDF 227 kb) [file 425_2025_4819_MOESM6_ESM.pdf]

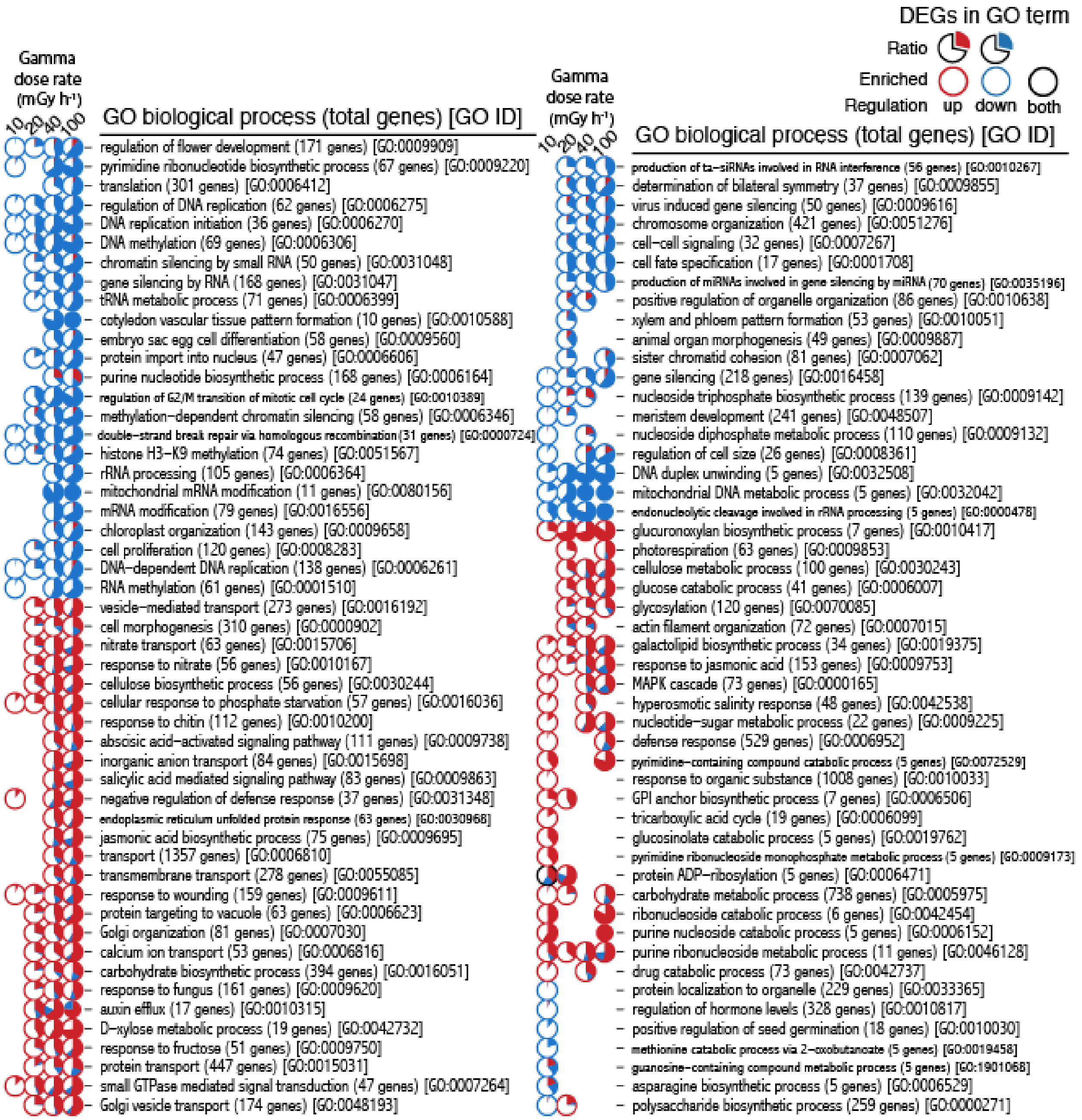

Figure S3. Bhattacherjee, Lee et al., 2025

Fig. S3 Gene Ontology (GO) enrichment analysis showing significant effects of 144-h irradiation with different gamma dose rates on expression of genes associated with various biological

processes in genetically identical stem cells of Norway spruce, relative to unexposed control cells. For each gamma dose rate, four repeated samples (*n=4*) were analysed in duplicate by RNA sequencing.
