# Supplementary material for: Gamma radiation-induced molecular toxicity and effects on pluripotent stem cells of the radiosensitive conifer Norway spruce (Picea abies)
Source: Planta. 2025 Sep 17;262(5):102. doi: 10.1007/s00425-025-04819-6 (PMC12443939; doi:10.1007/s00425-025-04819-6)
Supplement: Supplementary file 8 — Supplementary file8 (PDF 172 kb) [file 425_2025_4819_MOESM8_ESM.pdf]

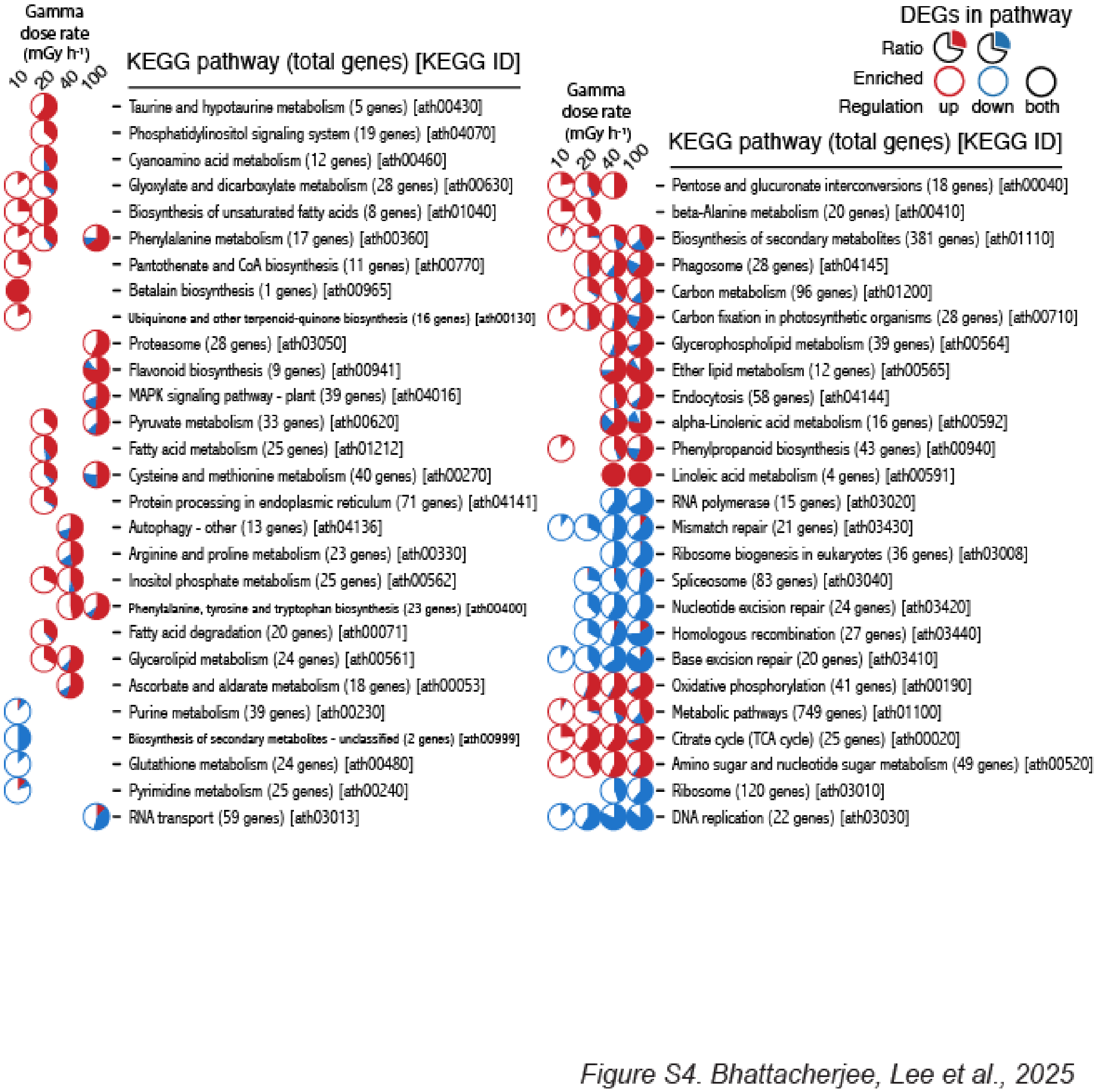

Fig. S4 KEGG ortholog enrichment and analysis of pathways (biological processes) significantly affected in genetically identical stem cells of Norway spruce exposed to different dose rates of gamma radiation for 144 h, relative to unexposed control cells. For each gamma dose rate, four repeated samples (*n=4*) were analysed in duplicate by RNA sequencing.
