# Supplementary material for: Gamma radiation-induced molecular toxicity and effects on pluripotent stem cells of the radiosensitive conifer Norway spruce (Picea abies)
Source: Planta. 2025 Sep 17;262(5):102. doi: 10.1007/s00425-025-04819-6 (PMC12443939; doi:10.1007/s00425-025-04819-6)
Supplement: Supplementary file 9 — Supplementary file9 (DOCX 16 kb) [file 425_2025_4819_MOESM9_ESM.docx]

**Table S1** Cell wall polysaccharide-directed monoclonal antibodies (MAb) used to study effect of 144-h of gamma irradiation on cell wall composition in stem cells of Norway spruce.

| **MAb** | **Cell wall polymer/epitope recognised** | **Reference** |
| --- | --- | --- |
| LM19 | Unesterified homogalacturonan | (Verhertbruggen et al. 2008) |
| LM20 | Methyl esterified homogalacturonan | (Verhertbruggen et al. 2008) |
| LM15 | XXXG motif of xyloglucan | (Marcus et al. 2008) |
| JIM13 | Arabinogalactan protein | (Yates and Knox 1994) |
