# Supplementary material for: Gamma radiation-induced molecular toxicity and effects on pluripotent stem cells of the radiosensitive conifer Norway spruce (Picea abies)
Source: Planta. 2025 Sep 17;262(5):102. doi: 10.1007/s00425-025-04819-6 (PMC12443939; doi:10.1007/s00425-025-04819-6)
Supplement: Supplementary file 10 — Supplementary file10 (DOCX 20 kb) [file 425_2025_4819_MOESM10_ESM.docx]

**Table S2.** Primers (F: forward, R: reverse) for qPCR analysis of DNA repair- and unfolded protein response (UPR)-related genes and for the housekeeping genes, after 144-h of gamma irradiation of stem cells of Norway spruce.

| Primer name | Sequence (5'-3') |
| --- | --- |
| DNA repair |  |
| *PaRAD5F* | GTGTGCCCAGTATCAATAGTTGGTC |
| *PaRAD5R* | CCTAGAGTGGCATATGTAGTAATCACTATG |
|  |  |
| *PaRAD50F* | GCGAGATAGATATATTCAAAGTGTGTTCGC |
| *PaRAD50R* | GCTTTTGATTTTCATGTCATTAGACTCCTTC |
|  |  |
| *PaXRCC3F* | CAGCTGCAATTGGGTCATTCTTC |
| *PaXRCC3R* | ATTTCCGATTGGCCTGAAGCCC |
|  |  |
| *PaGR1F* | GCTTCGGAGCTTGAGCGTGAAAG |
| *PaGR1R* | CCAGTGTCAGGTGCCTGATAATCG |
|  |  |
| *PaSOG1F* | CCACTACGTTCATGGAACCAAC |
| *PaSOG1R* | ATCCGGATCTGCAGCCTGTG |
|  |  |
| UPR |  |
| *PaHsp70F* | GAAATGGGAAAATGCAGCAAGAGCTTTC |
| *PaHsp70R* | CTGAATTTTCAATTACTTTTGGGTTCTTCCC |
|  |  |
| *PaHsp90F* | CAAGCACAACGACGATGAGCAATAC |
| *PaHsp90R* | CTCCTCCAAGTACTCCAGATGGTC |
|  |  |
| *PaRBRE3F* | CCAAGGAGTGTTGTAGCTTTGCATG |
| *PaRBRE3R* | GAATTCTGAGGTCCCTCCCCAAG |
|  |  |
| Housekeeping genes |  |
| *PaACTINF* | TGAGCTCCCTGATGGGCAGGTGA |
| *PaACTINR* | TGGATACCAGCAGCTTCCATCCCAAT |
|  |  |
| *Paα-TUBULINF* | CTGGAACCCACGGTCATT |
| *Paα-TUBULINR* | ACCACGAGCGAAGTTGTTG |
|  |  |
| *PaElongation Factor-1αF* | GGATTGCCACACTTGCCACA |
| *PaElongation Factor-1αF* | CTTGGGTTCCTTCTCCAGTTCC |
